# Geriatric nutritional risk index is associated with retinopathy in patients with type 2 diabetes

**DOI:** 10.1038/s41598-022-15463-5

**Published:** 2022-07-11

**Authors:** AJin Cho, Yun Soo Hong, Hayne Cho Park, Do Hyoung Kim, Young Joo Shin, Young-Ki Lee

**Affiliations:** 1 Department of Internal Medicine, Kangnam Sacred Heart Hospital, Hallym University College of Medicine, 1, Singil-ro, Yeongdeungpo-gu, Seoul, 07441 South Korea; 2 Hallym University Kidney Research Institute, Seoul, South Korea; 3 Department of Ophthalmology, Kangnam Sacred Heart Hospital, Seoul, Republic of Korea; 4 Departments of Epidemiology and Medicine, and Welch Center for Prevention, Epidemiology, and Clinical Research, Johns Hopkins University Bloomberg School of Public Health, Baltimore, MD USA

**Keywords:** Endocrinology, Health care, Medical research

## Abstract

The geriatric nutritional risk index (GNRI) is a nutrition-related risk assessment tool and has been used in various clinical settings. The relationship between body mass index (BMI) and the associated risk of diabetic retinopathy (DR) remains inconclusive. We aimed to evaluate the association between GNRI and DR in patients with type 2 diabetes. We included a total of 1359 patients with type 2 diabetes who followed up in our diabetes clinic and underwent fundus photographic examinations from August 2006 to February 2014. DR was assessed by retinal ophthalmologists using comprehensive ophthalmologic examinations. Patients were divided into tertiles according to their GNRI category. Patients in a lower GNRI tertile tended to have a higher proportion of nonproliferative DR (NPDR) and proliferative DR (PDR) compared with those in the other tertiles. The risk of PDR was higher in patients included in GNRI tertile 1 (Odds ratio (OR) 2.252, 95% Confidence Interval (CI) 1.080–4.823, *P* = 0.033) and GNRI tertile 2 (OR 2.602, 95% CI 1.323–5.336, *P* = 0.007) compared with those in GNRI tertile 3. In patients with lower GNRIs, the prevalence of DR was higher than in those with higher GNRIs. When GNRI was compared with BMI using the area under the curve, overall accuracy was high in GNRI. The risk of PDR was high in patients with low GNRI and there is an inverse association between GNRI scores and prevalence of DR. GNRI might be a useful tool to predict DR in patients with type 2 diabetes.

## Introduction

Diabetic retinopathy (DR) is the leading cause of preventable blindness in adults and is one of the major microvascular complications in patients with diabetes^1,2^. Hyperglycemia, hypertension, hyperlipidemia, and anemia contribute to the pathogenesis of DR through a series of pathological processes^3–5^. Current treatment modalities for DR include laser photocoagulation therapy, intravitreal corticosteroid and antivascular endothelial growth factor (VEGF) agent administration, and vitreo-retinal surgery^6–8^. These modalities are expensive and invasive and by the time they are administered, the retina has already undergone some damage. High prevalence of malnutrition is frequently unrecognized in patients with chronic disease and is associated with increased mortality and morbidity^9,10^. Despite controversy about timely and regular screening for nutritional problems in patients receiving health care, there is evidence that the use of simple screening procedures should be included in routine clinical practice as standard^9,11,12^. Nutritional deficiency plays an important role in diabetes-related complications such as nephropathy and diabetic foot ulcers^13,14^.

Nutrients can preserve retinal structure and function by interfering with the various pathological steps of DR incidence^15^. However, the complex interplay between nutrients and DR makes a nutritional therapy difficult to justify in having a major role in altering the risk of DR development. There have been several previous studies analyzing nutritional status in diabetic patients with DR that showed an association between body mass index (BMI) and obesity with DR^16–19^. However, the relationship between BMI and the associated risk of DR remained inconclusive because DR increases with uncontrolled diabetes, which also causes unintentional weight loss and a low BMI. Concurrently, obesity or a high BMI is often correlated with an escalating grade of DR.

The subjective global assessment (SGA) and the malnutrition inflammation score are commonly used to measure nutritional status^20,21^. However, both measurements need to be combined with subjective evaluation indicators. The geriatric nutritional risk index (GNRI), which is calculated using height, body weight, and serum albumin, was initially proposed to predict nutrition-related complications in hospitalized elderly patients^22^. Because of its simplicity, GNRI is widely applied in various clinical settings and has been associated with mortality not only in elderly patients but also in end-stage renal disease patients^23,24^. However, there are few data on the association between GNRI and diabetes-related complications. A recent study suggested that low GNRI is associated with a higher risk of sarcopenia which is associated with complications in patients with type 2 diabetes^25^. In this study, we assessed GNRI and DR status in patients with type 2 diabetes in our outpatient clinic. Non-hospitalized patients with chronic disease were included and we assessed GNRI as a nutrition screening tool in this patient population. We compared differences in clinical characteristics in the study subjects according to GNRI scores and investigated the association between GNRI and DR status.

## Materials and methods

### Study population

We enrolled 1359 patients with type 2 diabetes from the diabetes clinic in the Department of Endocrinology of Kangnam Sacred Heart Hospital who underwent fundus photographic examinations for DR and whose height, weight, and serum albumin levels were evaluated for GNRI assessment between August 2006 and February 2014. This observational study was performed in accordance with the Declaration of Helsinki and approved by the Institutional Review Board of Hallym University Kangnam Sacred Heart Hospital (IRB No: 2018-01-030). The written informed consent of the patients was waived by the Institutional Review Board of Hallym University Kangnam Sacred Heart Hospital because we used deidentified and retrospective data.

### Measurement

Baseline characteristics, including demographics (age, gender), medical history (diabetes, duration of diabetes), and laboratory variables were collected at the time of the first DR assessment. Patients were in a light clothing with a heavy jacket off for weight measurement. Their heights were obtained in a conventional way, standing height. Blood pressure was measured with a sphygmomanometer after 5 min of rest. Hemoglobin A1c (HbA1c) was measured using a method that was National Glycohemoglobin Standardization Program-certified and standardized to the Diabetes Control and Complications Trial assay. A standard urine dipstick was used to measure proteinuria qualitatively. Serum creatinine was measured using the modified Jaffe method. Based on the serum creatinine concentration, the estimated glomerular filtration rate (eGFR) was calculated using the four-variable equation from the Modification of Diet in Renal Disease study^26^.

### Determination of diabetic retinopathy

DR presence was assessed by retinal ophthalmologists who had no knowledge of the clinical details using slit-lamp examination, indirect ophthalmoscopy, and/or fluorescein angiography. Patients were classified into the following categories: (1) normal, no apparent sign of DR; (2) nonproliferative DR (NPDR), including microaneurysms, hard exudates, intraretinal hemorrhages, venous beading, or prominent intraretinal microvascular abnormality; and (3) proliferative DR (PDR), including retinal or optic disk neovascularization, vitreous hemorrhage, or preretinal hemorrhage, according to the Global Diabetic Retinopathy Project Group^27^. The presence and severity of DR in a participant were determined based on the eye showing the worst retinopathy.

### GNRI calculation method

The GNRI was calculated using the following equation^28^:

$${\text{GNRI }} = \, [{1}.{489} \times {\text{albumin }}\left( {{\text{g}}/{\text{L}}} \right)\left] { \, + \, } \right[{41}.{7} \times \left( {{\text{weight}}/{\text{ideal weight}}} \right)].$$

We set weight/ideal weight = 1 when the actual weight was greater than the ideal weight. The ideal weight was calculated from the Lorenz equation, as follows. For males, Height – 100 – [(height – 150)/4]. For females, Height – 100 – [(height – 150)/2.5].

### Statistical analyses

Data were expressed as mean (standard deviation) for continuous variables and as numbers of cases and percentages for categorical variables. Patients were stratified by DR status and GNRI tertiles. Differences between the groups were assessed using the chi-square test for dichotomous factors and one-way analysis of variance for continuous factors. Logistic regression analyses with stepwise variable selection were performed to assess the independent association between GNRI and DR. Univariate logistic regression models were employed first, followed by multivariate logistic regression models with adjustment by significant covariates (*P* < 0.05) in the univariate analysis. Receiver-operating characteristic (ROC) curves were analyzed to determine the overall accuracy of GNRI and BMI as measured by the area under the curve (AUC). We compared two models, containing GNRI and BMI respectively, with common parameters such as duration of diabetes, HbA1c and SBP. Differences between the ROC curves were tested with the DeLong test. All *P* values were two-sided, and *P* < 0.05 was considered statistically significant. Statistical analyses were performed using R version 4.0.5 (R Foundation for Statistical Computing, Vienna, Austria. URL https://www.R-project.org/).

### Institutional review board statement

This observational study was performed in accordance with the Declaration of Helsinki and approved by the Institutional Review Board of Hallym University Kangnam Sacred Heart Hospital (IRB No: 2018-01-030).

### Informed consent statement

The written informed consent of the patients was waived by the Institutional Review Board of Hallym University Kangnam Sacred Heart Hospital because we used deidentified and retrospective data.

## Results

### Baseline characteristics of the patients

**Figure 1:**
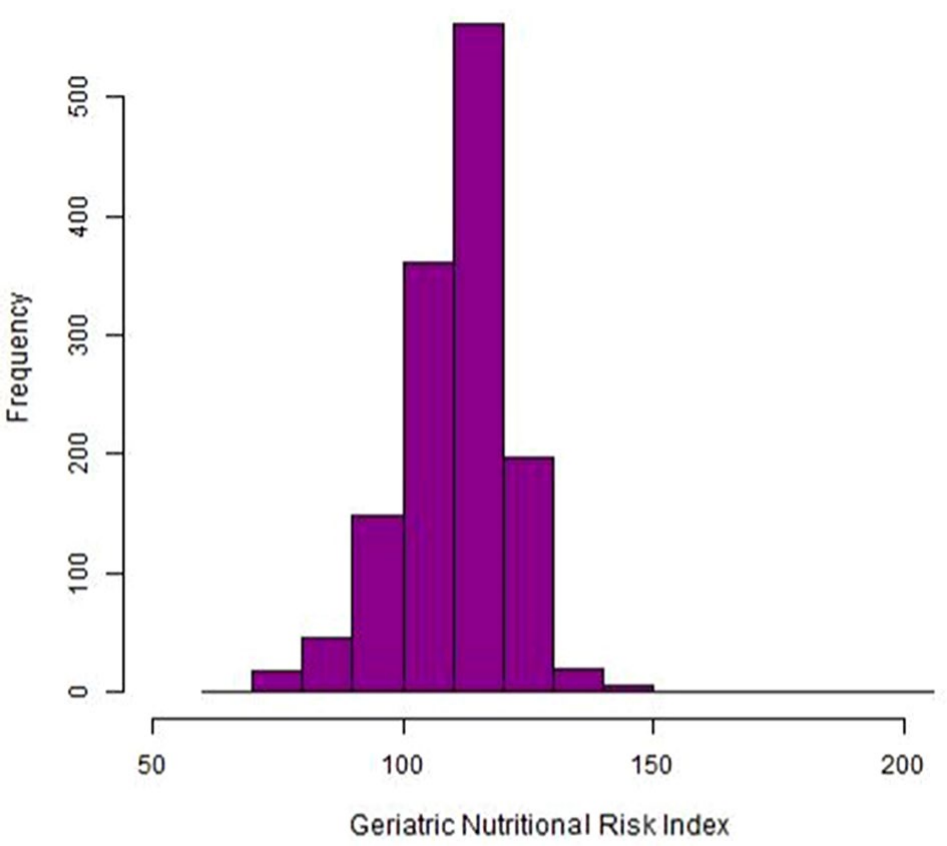
GNRI distribution of the study population.

Demographic and laboratory parameters of the patients according to retinopathy status are shown in Table 1. The mean age of all the patients was 58 years and 667 (49.1%) of them were men. The mean duration of diabetes was 8.6 years and increased as DR grade worsened. Systolic blood pressure (SBP), diastolic blood pressure, fasting plasma glucose (FPG), HbA1c, and proportion having proteinuria were higher in patients with DR. BMI, serum hemoglobin, and eGFR were decreased in patients with high grades of DR. The distribution of the GNRI in the study population is shown in Fig. 1. Table 2 shows comparisons of clinical and laboratory parameters by GNRI tertiles. The mean GNRI values of the tertiles were 98.3, 111.7, and 121.4. Patients in tertile 1 had a longer duration of diabetes and higher blood pressure as well as higher levels of FPG and HbA1c than those in the other tertiles. BMI and serum levels of hemoglobin, total cholesterol, and eGFR tended to decrease as patients were included in the lower tertiles.

**Table 1:** Baseline characteristics of the study population according to retinopathy status.

	All participants N = 1359	No DR N = 814	NPDR N = 335	PDR N = 210	P value
Age (years)	58 ± 11	58 ± 12	59 ± 11	56 ± 11	0.005
Male	667 (49.1)	396 (48.6)	164 (49.0)	107 (51.0)	0.836
Duration of diabetes (years)	8.6 ± 7.7	6.2 ± 6.4	11.6 ± 8.0	12.5 ± 8.3	< 0.001
BMI (kg/m^2^)	24.8 ± 3.7	25.2 ± 3.7	24.4 ± 3.7	23.6 ± 3.5	< 0.001
SBP (mmHg)	130.0 ± 19.7	127.7 ± 18.0	133.0 ± 21.3	134.1 ± 22.2	< 0.001
DBP (mmHg)	77.1 ± 13.0	76.1 ± 12.5	78.5 ± 13.8	79.1 ± 13.4	0.008
FPG (mg/dl)	154.4 ± 75.8	146.6 ± 68.3	164.2 ± 77.3	169.2 ± 94.8	< 0.001
HbA1c (%)	7.8 ± 1.9	7.5 ± 1.8	8.4 ± 2.0	8.3 ± 2.1	< 0.001
Hemoglobin (g/dl)	12.9 ± 2.0	13.4 ± 1.8	12.5 ± 1.9	11.7 ± 2.0	< 0.001
Total cholesterol (mg/dl)	167.4 ± 40.8	167.6 ± 36.6	166.1 ± 44.3	168.5 ± 49.5	0.778
eGFR (ml/min/1.73 m^2^)	73.6 ± 27.1	79.5 ± 23.6	69.7 ± 26.0	57.1 ± 33.0	< 0.001
**Proteinuria**					
–	1032 (84.2)	695 (93)	248 (79.7)	92 (53.8)	< 0.001
+–+++	194 (15.8)	52 (7)	63 (20.3)	79 (46.2)	

Data are expressed as mean ± standard deviation and number (percent).

DR, diabetic retinopathy; NPDR, nonproliferative diabetic retinopathy; PDR, proliferative diabetic retinopathy; BMI, body mass index; SBP, systolic blood pressure; DBP, diastolic blood pressure; FPG, fasting plasma glucose; Hemoglobin A1c, HbA1c; eGFR, estimated glomerular filtration rate.

**Table 2:** Comparison of clinical and laboratory parameters by GNRI tertiles.

	GNRI tertiles			P value
	T1 (n = 453)	T2 (n = 453)	T3 (n = 453)	
Age (years)	59 ± 12	59 ± 11	56 ± 11	< 0.001
Male	227 (50.1)	230 (50.8)	210 (46.4)	0.358
Duration of diabetes (years)	10.3 ± 8.5	8.8 ± 7.5	6.6 ± 6.6	< 0.001
BMI (kg/m^2^)	22.2 ± 2.8	24.4 ± 2.2	27.8 ± 3.5	< 0.001
SBP (mmHg)	130.7 ± 22.1	129.5 ± 18.9	129.7 ± 17.5	0.705
DBP (mmHg)	77.5 ± 13.9	76.9 ± 12.9	76.9 ± 12.1	0.776
FPG (mg/dl)	164.3 ± 90.2	152.6 ± 72.3	146.4 ± 60.8	0.002
HbA1c (%)	8.5 ± 2.4	7.6 ± 1.6	7.5 ± 1.5	< 0.001
Hemoglobin (g/dl)	11.8 ± 2.0	13.3 ± 1.6	13.7 ± 1.7	< 0.001
Total cholesterol (mg/dl)	161.6 ± 47.7	167.1 ± 35.4	173.4 ± 37.3	< 0.001
eGFR ml/min/1.73 m^2^)	69.1 ± 33.6	73.9 ± 23.2	77.9 ± 22.4	< 0.001
**Proteinuria**				
−	285 (68.5)	365 (89.5)	385 (95.1)	< 0.001
+–+++	131 (31.5)	43 (10.5)	20 (4.9)	
GNRI	98.3 ± 7.7	111.7 ± 2.2	121.4 ± 6.8	< 0.001

Data are expressed as mean ± standard deviation and number (percent).

DR, diabetic retinopathy; NPDR, non-proliferative diabetic retinopathy; PDR, proliferative diabetic retinopathy; BMI, body mass index; SBP, systolic blood pressure; DBP, diastolic blood pressure; FPG, fasting plasma glucose; Hemoglobin A1c, HbA1c; eGFR, estimated glomerular filtration rate; GNRI, geriatric nutritional risk index.

### Prevalent diabetic retinopathy and GNRI tertiles

**Figure 2:**
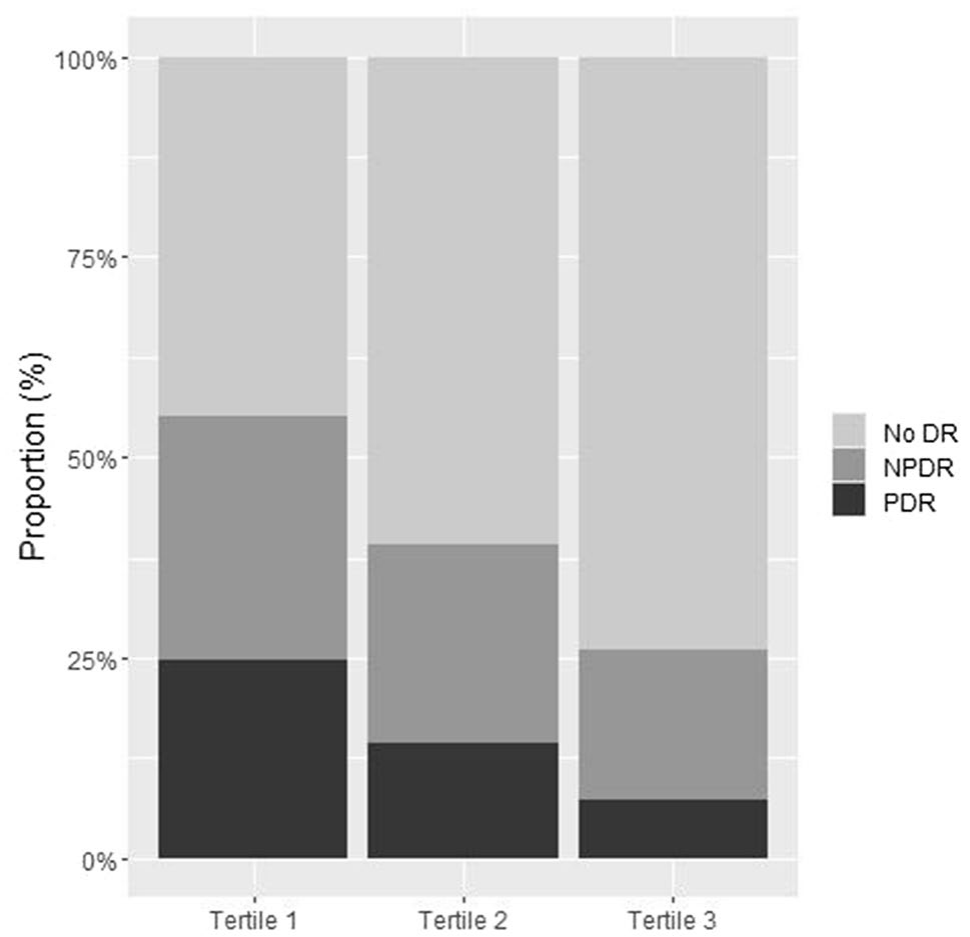
Retinopathy proportion according to GNRI tertiles.

The prevalence rate of DR according to GNRI tertiles is shown in Fig. 2. The number of patients with DR was 250 (55.2%) in tertile 1, 177 (39.1%) in tertile 2, and 118 (26%) in tertile 3. Patients in tertile 1 tended to have a higher proportion of NPDR and PDR than those in the other tertiles (*P* < 0.001).

### Association between diabetic retinopathy and GNRI

**Figure 3:**
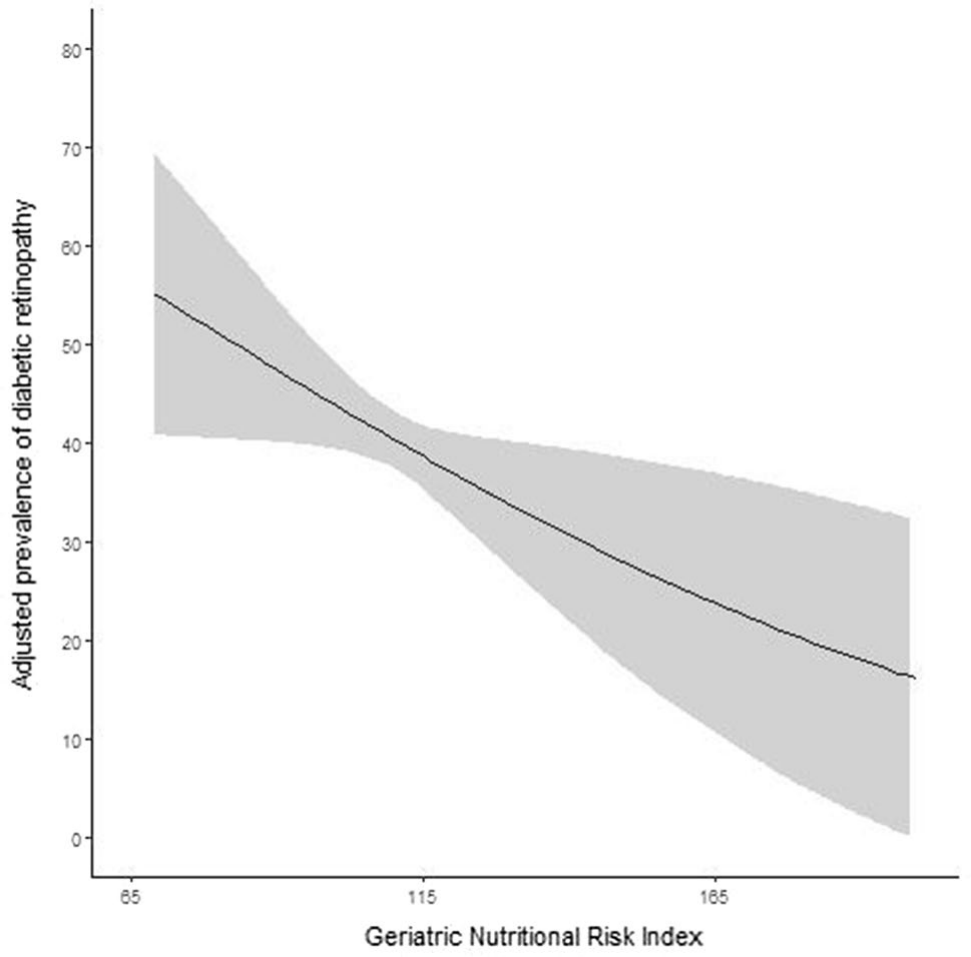
Adjusted prevalence of diabetic retinopathy.

We performed multivariate logistic regression analyses and NPDR and PDR were considered as dependent variables (Table 3). Longer duration of diabetes (Odds ratio (OR) 1.100, 95% Confidence Interval (CI) 1.070–1.132, *P* < 0.001), higher HbA1c (OR 1.289, 95% CI 1.159–1.440, *P* < 0.001), lower hemoglobin (OR 0.853, 95% CI 0.758–0.958, *P* = 0.008), and higher SBP (OR 1.013, 95% CI 1.003–1.023, *P* = 0.009) were associated with the presence of NPDR in diabetes patients. The risk of PDR was higher in patients of younger age (OR 0.950, 95% CI 0.926–0.974, *P* < 0.001), longer duration of diabetes (OR 1.106, 95% CI 1.067–1.148, *P* < 0.001), higher HbA1c (OR 1.171, 95% CI 1.015–1.351, *P* = 0.030), lower hemoglobin (OR 0.687, 95% CI 0.582–0.806, *P* < 0.001), proteinuria (OR 2.717, 95% CI 1.339–5.431, *P* = 0.005), and being in tertile 1 (OR 2.252, 95% CI 1.080–4.823, *P* = 0.033) and tertile 2 (OR 2.602, 95% CI 1.323–5.336, *P* = 0.007) of the GNRI scores. Figure 3 shows adjusted marginal prevalence of DR. For patients with lower GNRI, the prevalence of DR was higher than for those with higher GNRI.

**Table 3:** The association of GNRI categories with diabetic retinopathy.

Variables	Multivariate logistic regression models			
	NPDR vs. no DR		PDR vs. no DR	
	Odds ratio (95% CI)	P value	Odds ratio (95% CI)	P value
Age (per year)	0.996 (0.977–1.015)	0.657	0.950 (0.926–0.974)	< 0.001
Duration of diabetes (per year)	1.100 (1.070–1.132)	< 0.001	1.106 (1.067–1.148)	< 0.001
FPG (per mg/dl)	1.001 (0.998–1.004)	0.346	1.000 (0.996–1.004)	0.925
HbA1c (per %)	1.289 (1.159–1.440)	< 0.001	1.171 (1.015–1.351)	0.030
Hemoglobin (per g/dl)	0.853 (0.758–0.958)	0.008	0.687 (0.582–0.806)	< 0.001
SBP (per mmHg)	1.013 (1.003–1.023)	0.009	1.011 (0.998–1.023)	0.109
Proteinuria (vs. no)	1.687 (0.921–3.069)	0.088	2.717 (1.339–5.431)	0.005
eGFR (per ml/min/1.73 m^2^)	0.994 (0.985–1.002)	0.157	0.991 (0.980–1.002)	0.104
Tertile 1 (vs. tertile 3)	1.489 (0.889–2.464)	0.121	2.252 (1.080–4.823)	0.033
Tertile 2 (vs. tertile 3)	1.095 (0.694–1.729)	0.696	2.602 (1.323–5.336)	0.007

SBP, systolic blood pressure; FPG, fasting plasma glucose; Hemoglobin A1c, HbA1c; eGFR, estimated glomerular filtration rate; GNRI, geriatric nutritional risk index.

### Comparison of GNRI with BMI

We assessed the accuracy of GNRI and BMI as a measurement for DR risk and compared the two measurements using AUCs. AUCs of model with GNRI and BMI were 80.2 and 78.7, respectively, and the difference was significant (*P* = 0.007).

## Discussion

In this study, we investigated the association between nutrition status and DR in patients with type 2 diabetes. We used GNRI, a reliable nutrition screening tool. The results of the present study showed that a low GNRI was significantly associated with a risk of PDR and the adjusted prevalence of any DR tended to be high when a patient had a low GNRI. Further, we did multivariate logistic regression analyses for PDR with interaction of chronic kidney disease (CKD) which was defined as eGFR less than 60 ml/min/1.73 m^2^. P values of CKD interactions were not significant at ORs of GNRI Tertile 1 vs Tertile 3 (p-value = 0.380) and GNRI Tertile 2 vs Tertile 3 (p-value = 0.980). The association between low GNRI and PDR risk was significant independently of CKD status.

Nutrition strategies can reduce the risk of developing DR and preserve the normal physiology and structure of the retina in patients with type 2 diabetes. As current treatments are invasive and expensive, a nutrition-based approach can be an adjunct therapy inhibiting the development and progression of DR^29^. SGA is a reliable clinical assessment method of nutritional status, based on the medical history and physical examination of the 130,677 subject providing a thorough estimation of nutritional status^29^. Previous studies showed that SGA scores were correlated with the presence and severity of DR status^30,31^. SGA scores are calculated after evaluating overall health status but include subjective factors to assess nutritional status.

We used the GNRI score, which combines two nutritional indicators, albumin and actual weight compared with desirable weight. It has been reported that acute and chronic inflammation contribute to hypoalbuminemia, and inflammation is involved in the pathogenesis of chronic diabetic complications^32^. BMI is an important indicator of nutrition status and a measure of obesity. Therefore, a combination of BMI with albumin could be a good index in evaluating nutrition status and predicting clinical outcomes and mortality in patients with a medical disease. A recent study has shown that GNRI is associated with renal progression and cardiovascular disease in patients with chronic kidney disease^14^. In diabetic foot patients, GNRI independently predicted mortality^13^.

Obesity has been considered as a risk factor for DR in several studies^18,19,33^. BMI is a commonly used measure to assess and manage obesity^34^. Obesity or high BMI is often correlated with the progression of DR, and this can be explained by the fact that obesity increases inflammation, oxidative stress, and insulin resistance^35,36^. Obesity also features hypertension and hyperlipidemia, which are contributing factors of DR^37^. Many studies found an association between BMI and obesity with DR but the relationship between BMI and the associated risk of DR remained inconclusive^38^. Some studies have shown a positive or negative association between BMI and DR, but others demonstrated no statistically significant relationship^18,19,33,39,40^. This inconsistency may be attributed to differences in study design, participant characteristics, and race or ethnicity.

The Asian Eye Epidemiology Consortium conducted a cross-sectional pooled analysis in 12 Asian populations with diabetes to clarify the association between BMI and DR in Asians^41^. They found an inverse relationship between obesity and DR. We used GNRI as an indicator of nutrition status and the findings show that a low GNRI is associated with the presence and severity of DR and thus confirms the results of the majority of previously conducted studies in Asian populations^19,33^. Meanwhile, studies conducted in Western populations reported either a positive or a null association between BMI and DR^39,40,42^. It has been shown that at the same BMI, Asians tend to have a higher risk of adverse clinical outcomes than Western populations in association with a differential body fat distribution^43^. However, further study should be conducted to evaluate the association between obesity and DR in different races. The exact mechanism underlying the inverse association between BMI and DR is not clear. Some possible explanations are survival bias, which means that those with obesity and severe DR might have died earlier, and the genetic predisposition to type 2 diabetes being stronger in lean than in obese patients with type 2 diabetes.

We evaluated the accuracy of GNRI and BMI in assessing the risk of DR and compared the two indicators using AUCs. The GNRI has a higher AUC than BMI and the difference was significant. BMI measurements have shown inconsistent results in previous studies of DR and hypoalbuminemia is associated with inflammation in patients with type 2 diabetes^32^. Moreover, diabetes-related complications are complex diseases and are affected by multiple factors. In this regard, the GNRI is an integrated expression of its components; thus, it might be a more practical indicator of a patient’s nutrition status and clinical outcomes than BMI. Many chronic diseases are associated with sarcopenia which means loss of skeletal muscle mass and strength^44^. A recent study showed sarcopenia is associated with PDR in patients with type 2 diabetes^45^. In a Japanese cohort study, GNRI is related to presence of sarcopenia in this population^46^. To our knowledge, this study is the first in which the association of DR with nutritional status using GNRI has been investigated in a population with diabetes. Further study including other races and larger numbers of participants should be conducted to clarify our results.

There are some limitations to our study. First, there is the inherent weakness of all studies with a cross-sectional design. Thus, we cannot propose a causal association or prediction of GNRI scores with developing DR in patients with type 2 diabetes. Second, we did not include waist circumference (WC) measurement to complete anthropometric profile of the study subjects, since WC is associated with DR. Third, DR classification in this study was based on graders’ discretion, which might result in potential bias. However, each experienced retinal specialist determined DR grades according to globally accepted guidelines. Fourth, our study was performed only in the Korean population, which might not be generalizable to other populations. Therefore, the predictive validity of the GNRI needs to be examined in patients with diabetes from other countries of origin.

## Conclusions

Our findings showed an inverse association between GNRI scores and DR. The GNRI might be a useful tool to predict DR in patients with type 2 diabetes. To confirm our findings, longitudinal studies based on different races should be conducted to determine the association between nutrition status using GNRI and DR, and evaluate the effect of weight change on the development and progression of DR.

## Data Availability

The data presented in this study are available on request from the corresponding author.
